# Effect of Multimodal Pre-emptive Analgesia with Pregabalin Versus Naproxen on Post-Thoracotomy Pain and Opioid Consumption: A Randomized Clinical Trial

**DOI:** 10.4274/TJAR.2026.262403

**Published:** 2026-08-28

**Authors:** Prajjal Das, Rajni Gupta

**Affiliations:** 1 Uttar Pradesh University of Medical Sciences, Department of Anaesthesiology, Saifai, India; 2 King George Medical University, Department of Anaesthesiology, Lucknow, India

**Keywords:** Analgesia, analgesics, epidural, naproxen, pain, pregabalin, thoracotomy

## Abstract

**Objective:**

Thoracotomy produces severe postoperative pain that limits deep breathing and coughing and may worsen recovery. Opioid-centred regimens can cause adverse effects; pre-emptive multimodal analgesia may reduce central sensitisation and opioid use.

**Methods:**

In this randomized, active-controlled, assessor-blinded trial (CTRI/2022/07/043800), adults aged 18-70 years (American Society of Anesthesiologists I-II) undergoing thoracotomy were randomized (39 per group) to receive oral pregabalin 2.5 mg kg^-1^ or oral naproxen 7 mg kg^-1^ (maximum 500 mg) 2 hours preoperatively. All patients received standardized general anaesthesia combined with thoracic epidural analgesia. Numerical rating scale (NRS) pain at rest and during deep breathing and coughing were assessed at 2, 6, 12, and 24 hours. A rescue opioid was administered when NRS exceeded 3. Time to first rescue, total 24-hour opioid consumption, number of rescue doses, sleep interference, and adverse events were recorded.

**Results:**

Baseline demographics and perioperative haemodynamics were comparable. Resting pain scores and sleep interference were similar between groups. Pregabalin reduced opioid requirement: fewer patients required rescue analgesia (46.2% vs. 69.2%; *P*=0.042), time to first rescue was longer (7.6±3.1 vs. 5.4±2.8 h; *P*=0.001), and 24-hour opioid consumption was lower (6.2±3.0 vs. 8.6±3.4 mg morphine equivalents; *P*=0.001). Dynamic pain during deep breathing and coughing was lower with pregabalin at early time points; sedation was slightly higher at 2 hours, without significant adverse neurocognitive events.

**Conclusion:**

Pre-emptive pregabalin improved functional analgesia and produced an opioid-sparing effect compared with naproxen after thoracotomy, with acceptable short-term safety, thereby supporting enhanced recovery pathways.

**Main Points**

• Pre-emptive pregabalin and naproxen provided effective control of postoperative pain at rest after thoracotomy when administered as part of a multimodal analgesic regimen that included thoracic epidural analgesia.

• Pregabalin demonstrated a significant opioid-sparing effect, with fewer patients requiring rescue analgesia, longer time to first rescue dose, lower total opioid consumption, and fewer rescue doses within the first 24 hours postoperatively.

• Functional analgesia was slightly better with pregabalin, as evidenced by significantly lower pain scores during deep breathing and coughing in the early postoperative period. This is clinically important for respiratory mechanics and pulmonary recovery. No major safety concerns were identified during the study period.

## Introduction

Thoracotomy was a commonly performed surgical approach that involved an incision through the intercostal space to access the thoracic cavity and was indicated for a wide range of cardiothoracic and pulmonary conditions, including distal aortic, cardiac, esophageal, and pulmonary diseases such as primary or metastatic lung malignancies, pleural tumors, pneumothorax, and empyema.^1, 2, 3^ In the present study, the majority of patients who underwent thoracotomy had empyema thoracis. Despite advances in surgical and anaesthetic techniques, thoracotomy remains among the most painful surgical procedures, with significant implications for postoperative recovery and morbidity.

Acute pain functioned as a protective physiological response; however, postoperative pain represented a maladaptive phenomenon that conferred no benefit and adversely affected multiple organ systems.^4^ Inadequately controlled postoperative pain has been associated with prolonged hospital stay, an increased risk of pulmonary and wound infections, delayed mobilisation, and impaired functional recovery. Moreover, acute postoperative pain was a strong predictor of chronic post-surgical pain, which is reported in 10-65% of patients, underscoring the importance of effective perioperative analgesic strategies.^5, 6^ Optimal pain control was therefore essential to enhance patient satisfaction, facilitate early ambulation and respiratory physiotherapy, reduce hospitalisation, and prevent immobility-related complications.^7^

Post-thoracotomy pain, reported in 20-80% of patients, exerts a profound negative impact on surgical outcomes by triggering sympathetic overactivity, resulting in tachycardia, hypertension, myocardial ischaemia, and reduced alveolar ventilation, and by impairing wound healing and causing significant patient discomfort. Although multimodal analgesia incorporating opioids and non-steroidal anti-inflammatory drugs (NSAIDs) was routinely practiced, opioid-based regimens were frequently limited by adverse effects such as nausea, vomiting, pruritus, and respiratory depression, and were often inadequate in achieving optimal analgesia.^8, 9, 10^ These limitations provided a strong rationale for the use of pre-emptive analgesia, wherein analgesic agents were administered prior to the surgical insult to attenuate central sensitisation, hyperalgesia, and allodynia.

Surgical tissue injury led to the release of inflammatory mediators, including prostaglandins and bradykinin, which activated peripheral nociceptors and transmitted pain signals through the dorsal horn of the spinal cord, with N-methyl-D-aspartate receptor pathways playing a critical role in pain amplification and persistence.^11, 12, 13^ Multimodal analgesia targeted multiple components of this pain pathway to achieve superior analgesic outcomes.^14^ Pregabalin, by binding to the α2δ subunit of presynaptic voltage-gated calcium channels, reduced the release of excitatory neurotransmitters, while naproxen inhibited cyclooxygenase-1 and -2 enzymes, thereby decreasing prostaglandin synthesis and limiting both peripheral and central sensitisation.^15, 16^ Owing to limited direct comparative evidence between these agents as components of pre-emptive multimodal analgesia in thoracotomy, the present randomized study was undertaken to evaluate and compare the effects of pre-emptive pregabalin versus naproxen on postoperative pain intensity, functional pain, and opioid consumption in patients undergoing thoracotomy.

## Methods

### Study Design and Setting

This study was conducted as a prospective, randomized, active-controlled, assessor-blinded trial at a tertiary care centre in Northern India. The study was carried out in the Department of Anaesthesiology, in collaboration with the Department of General Surgery. The duration of the study was one year. Ethical approval was obtained from the Institutional Ethics Committee of King George’s Medical University, U.P., (approval no: VI-PGTSC-IIA/P29, date: 27.10.2021). The study was registered in the Clinical Trials Registry of India (CTRI/2022/07/043800). Written informed consent was obtained from all participants prior to enrolment.

### Study Participants

Adult patients of either sex who were scheduled to undergo thoracotomy were screened for eligibility. Surgical procedures included lobectomy, pneumonectomy, wedge resection, and decortication (specify the actual procedures and their numbers). Thoracoscopic (video-assisted thoracoscopic surgery) procedures were excluded. The thoracotomy incision was made in the fifth or sixth intercostal space, according to surgical indication. Inclusion criteria were patients aged 18-70 years, with American Society of Anesthesiologists (ASA) physical status I or II, and normal renal function (creatinine clearance >30 mL min^-^1). Exclusion criteria were: use of opioids, antidepressants, or anticonvulsants prior to surgery; presence of serious, uncontrolled systemic illness (cardiac, pulmonary, hepatic, renal, or endocrine disorders); pregnancy or lactation; history of angioedema or drug hypersensitivity; and contraindications to epidural analgesia such as spinal deformity or anticoagulant therapy.

Sample size was calculated based on a previous study (Ethemoglu and Calik^17^), where the mean difference in postoperative day 1 numerical rating scale (NRS) between epidural analgesia and pregabalin groups was 1.17 (4.64 vs. 3.47), with variance (σ)=1.85. With α=0.05 (Zα/2=1.96) and a power of 80% [Z(1-β)=0.84], the computed sample size was 39 patients per group.

### Intervention Groups

Participants were divided into two groups:

• **Group I **(Pregabalingroup): Patients received oral pregabalin at 2.5 mg kg^-1^, administered 2 hours before surgery.

• **Group II **(Naproxengroup): Patients received oral naproxen at 7 mg kg^-1 ^(or a fixed dose of 500 mg as per protocol), administered 2 hours before surgery.

Both groups received standardized multimodal general anaesthesia and thoracic epidural analgesia.

### Randomization, Allocation, Concealment, and Blinding

Randomization was performed using a computer-generated random-number sequence to allocate patients to either Group I or Group II. Allocation was performed prior to surgery. Outcome assessment was carried out by a trained investigator who was blinded to group allocation. Patients were instructed not to disclose the study medication to the assessor to maintain assessor blinding. Patient blinding was not performed because of the nature of the intervention; therefore, the study was conducted as an assessor-blinded trial.

### Intervention and Data Collection Process

Eligible patients scheduled for thoracotomy were screened at the pre-anaesthetic evaluation clinic, and those fulfilling the inclusion criteria were enrolled after obtaining written informed consent. Enrolled patients were admitted to the surgical ward one day prior to surgery, where baseline demographic details, clinical history, ASA physical status, and routine preoperative investigations were recorded. On the day of surgery, patients received the allocated pre-emptive study medication orally with sips of water, approximately two hours before induction of anaesthesia; Group I received pregabalin at 2.5 mg kg^-1^, while Group II received naproxen at 7 mg kg^-1 ^or a fixed dose of 500 mg, as per protocol.

In the operating theatre, standard monitoring was established, following which a thoracic epidural catheter was inserted at the T7-T9 intervertebral level before induction of general anaesthesia. The epidural space was identified using the loss-of-resistance technique, and the catheter was advanced 6-8 cm into it. Correct placement was confirmed by assessment of sensory blockade using cold stimulation. Epidural analgesia was initiated at the start of surgery by administering 10 mL of 0.2% ropivacaine combined with dexmedetomidine (0.5 μg kg^-1^) through the epidural catheter. Thereafter, all patients were induced and maintained under general anaesthesia with a standardized multimodal anaesthetic technique. Intraoperative hemodynamic parameters were recorded at predefined intervals. Total epidural local anaesthetic consumption and rescue epidural boluses were not recorded, which limits the interpretation of the independent analgesic contribution of pregabalin.

Postoperatively, patients were shifted to the recovery area and subsequently to the ward for continued monitoring. Pain intensity was assessed using the NRS at 2, 6, 12, and 24 hours after surgery, both at rest and during movement, including deep breathing and coughing. The sleep interference rate (SIR) was assessed on the morning of postoperative day one. Rescue analgesia was administered whenever the NRS score exceeded 3; the time to first rescue analgesic, total opioid consumption in the first 24 hours, and number of rescue doses required were documented. Patients were monitored throughout the postoperative period for adverse effects such as sedation, dizziness, somnolence, nausea, vomiting, confusion, and visual disturbances.

Follow-up continued for 24 hours postoperatively, during which all pain scores, sleep interference, analgesic requirements, and adverse events were systematically recorded and entered into the study pro forma for final analysis.

### Statistical Analysis

Data were analysed using SPSS version 26.0. Data were checked for normality before analysis. Continuous variables were expressed as mean ± standard deviation for normally distributed data and as median with interquartile range for non-normally distributed data. Categorical variables were expressed as frequencies and percentages. Intergroup comparisons of baseline continuous variables were performed using the Student’s t-test or the Mann-Whitney U test, as appropriate; categorical variables were compared using the chi-square test or Fisher’s exact test, as appropriate. Postoperative pain and sedation scores, measured repeatedly over time, were analysed using repeated-measures analysis of variance, with group as the between-subjects factor and time as the within-subjects factor. The group × time interaction was assessed to evaluate whether changes in scores over time differed between the study groups. Bonferroni-adjusted post hoc analyses were performed for multiple comparisons whenever significant main or interaction effects were observed. A *P* value of <0.05 was considered statistically significant.

## Results

The baseline demographic and clinical characteristics were comparable between the two groups, with a similar mean age (37.72±13.92 years in Group I vs. 38.49±15.69 years in Group II), gender distribution, and ASA physical status, indicating adequate baseline comparability (Table 1). Perioperative hemodynamic parameters, including heart rate and diastolic blood pressure, remained comparable at all measured time points. A transient but statistically significant difference in systolic blood pressure was observed at 15 minutes (119.59±8.41 mmHg in Group I vs. 124.08±10.39 mmHg in Group II; *P*=0.039), which was not sustained at later intervals and was not clinically significant (Table 2).

Postoperative pain scores at rest were low and comparable between the two groups at all time points, with no statistically significant differences at 2, 6, 12, or 24 hours (*P* > 0.05 for all). SIR were also similar, with SIR-01 reported in 30.8% of patients in Group I and 35.9% of patients in Group II, SIR-02 in 46.2% of patients in Group I and 43.6% of patients in Group II, and SIR-03 in 23.1% of patients in Group I and 20.5% of patients in Group II, indicating comparable sleep quality in both groups (Table 3).

This enhanced analgesic efficacy translated into a significant opioid-sparing effect in Group I, with fewer patients requiring rescue analgesia (46.2% vs. 69.2%; *P*=0.042),a longer time to first rescue dose (7.6±3.1 vs. 5.4±2.8 hours; *P*=0.001),and lower total opioid consumption in the first 24 hours (6.2±3.0 mg vs. 8.6±3.4 mg morphine equivalents; *P*=0.001),along with fewer rescue doses [median 1 (0-2) vs. 2 (1-3); *P*=0.008] (Table 4).

In contrast, Group I demonstrated better control of movement-related pain. Pain scores on deep breathing were significantly lower in Group I at 2 hours (2.64±0.91 vs. 3.08±0.88; *P*=0.033) and 6 hours (2.18±0.84 vs. 2.62±0.79; *P*=0.019), while pain on coughing was significantly lower at 2 hours (3.10±0.96 vs. 3.74±0.93; *P*=0.004), 6 hours (2.72±0.89 vs. 3.26±0.90; *P*=0.010), and 12 hours (2.33±0.78 vs. 2.71±0.82; *P*=0.040), reflecting improved functional analgesia in Group I (Table 5).

Sedation scores were marginally higher in Group I at 2 hours postoperatively (2.38±0.54 vs. 2.10±0.45; *P*=0.015), but this difference was not observed at 6 hours. The incidence of excessive sedation, dizziness, somnolence, confusion, and visual disturbances was numerically higher in Group I, but the difference did not reach statistical significance, which indicates an acceptable safety and neurocognitive profile for both regimens (Table 6).

## Discussion

Effective management of post-thoracotomy pain is essential, as inadequate analgesia adversely affects respiratory mechanics, delays mobilization, and increases the risk of pulmonary complications and chronic post-thoracotomy pain syndrome. Contemporary pain management strategies emphasize opioid-sparing multimodal analgesia to optimize recovery and reduce opioid-related adverse effects. In the present study, both pregabalin and naproxen provided satisfactory control of resting pain during the early postoperative period. However, clinically meaningful differences were observed with respect to opioid requirement, functional pain control, and overall analgesic quality.^18, 19^

In our study, baseline demographic and perioperative variables were comparable between the pregabalin and naproxen groups, ensuring a valid comparison of analgesic outcomes. Postoperative pain scores at rest and rates of sleep interference were similar across groups at all assessed time points, indicating that both drugs effectively controlled baseline nociceptive pain. This finding is consistent with previous evidence supporting non-opioid agents as effective components of multimodal analgesia in thoracic surgery.

However, the pregabalin group demonstrated a significant opioid-sparing effect compared with the naproxen group. Fewer patients in the pregabalin group required rescue analgesia; the time to the first rescue dose was significantly longer; and total opioid consumption in the first 24 hours was substantially lower. These findings align with earlier studies by Matsutani et al.,^20^ who reported reduced analgesic requirements and improved pain-related outcomes with pregabalin compared with epidural analgesia, and with meta-analytic evidence demonstrating reduced acute postoperative pain scores and opioid use following thoracic surgery when pregabalin is incorporated into the analgesic regimen.

Importantly, pregabalin was associated with better control of movement-related pain. Pain scores during deep breathing and coughing were significantly lower in the pregabalin group, particularly during the early postoperative hours. This observation is clinically relevant because effective analgesia during respiratory maneuvers is critical after thoracotomy for facilitating chest expansion, improving cough efficacy, and reducing pulmonary complications. Naproxen, while effective for inflammatory pain, primarily inhibits peripheral cyclooxygenase and may be less effective at modulating central sensitization and the neuropathic components of post-thoracotomy pain. In contrast, pregabalin acts centrally by binding to the α2δ subunit of voltage-gated calcium channels, reducing excitatory neurotransmitter release and attenuating central sensitization, which likely explains its better performance in functional pain control.^21^

Our findings are in agreement with Mishra et al.,^22^ who demonstrated that pregabalin significantly reduced chronic post-thoracotomy pain at longer follow-up intervals, although acute pain scores were comparable initially. While our study did not extend into long-term follow-up, improved early functional analgesia and reduced opioid consumption may play a role in preventing central sensitization and the subsequent development of persistent pain syndromes.^22^

Contrasting evidence from non-thoracic surgical populations must also be considered. Choi et al.^23^reported no significant analgesic advantage of pregabalin over NSAIDs following ankle fracture fixation, with a higher incidence of dizziness and somnolence in the pregabalin group. This discrepancy highlights that the analgesic benefit of pregabalin is procedure-specific and may be more pronounced in surgeries such as thoracotomy, where neuropathic and centrally mediated pain components are prominent.^23^

Regarding safety, the pregabalin group in our study exhibited slightly higher sedation scores in the immediate postoperative period; however, this effect was transient and not associated with clinically significant adverse events such as confusion, excessive somnolence, or visual disturbances. Although dizziness and somnolence have been reported with pregabalin in other studies,^24, 25^ the incidence in our cohort did not differ significantly from that observed in the naproxen group, indicating an acceptable safety profile at the administered dose of 2.5 mg kg^-1^.

The effectiveness of naproxen as part of multimodal analgesia is well supported, as highlighted by Weisman^26^ who demonstrated its efficacy in reducing postoperative pain and opioid consumption without increasing bleeding risk. The comparable pain scores at rest observed in our naproxen group likely reflect this benefit. However, the more effective opioid-sparing effect and improved dynamic pain control with pregabalin suggest that centrally acting agents may provide additional advantages in thoracic surgical patients.

While both pregabalin and naproxen were effective in controlling postoperative pain at rest following thoracotomy, pregabalin was associated with better pain control during coughing and deep breathing and with reduced postoperative opioid consumption. These findings suggest that pregabalin may be a valuable component of a multimodal analgesic regimen for thoracic surgery; however, larger multicentre studies are warranted to confirm these observations.

A key strength of this study is its randomized design with comparable baseline characteristics, allowing a reliable comparison between pregabalin and naproxen as pre-emptive analgesic agents and a comprehensive assessment of pain at rest, functional pain, opioid consumption, and adverse effects. However, the study is limited by its relatively short follow-up duration, which precludes assessment of chronic post-thoracotomy pain, and by a modest sample size, limiting generalizability; additionally, the absence of patient-reported quality-of-life measures and objective pulmonary outcomes represents a further limitation; by a modest sample size, which limits generalizability; and by the absence of patient-reported quality-of-life measures and objective pulmonary outcomes.

### Study Limitations

This study was limited by its single-center design, relatively small sample size, and lack of long-term follow-up for chronic post-thoracotomy pain. Pain assessment was based on subjective scoring systems, and the lack of clearly defined blinding procedures may have introduced bias. Different doses of pregabalin were not evaluated. As all patients received thoracic epidural analgesia, the observed benefit of pregabalin should be interpreted as an additive effect within a multimodal analgesic regimen rather than as evidence of its independent superiority. Total epidural local anaesthetic consumption and rescue epidural boluses were not recorded, which limits interpretation of the independent analgesic contribution of pregabalin.

## Conclusion

Both pregabalin and naproxen provided effective control of postoperative pain at rest following thoracotomy. Pre-emptive pregabalin was associated with improved functional analgesia during deep breathing and coughing, and with reduced postoperative opioid consumption, without a statistically significant increase in adverse events. These findings suggest that pregabalin may offer an additive benefit as part of a multimodal analgesic regimen for thoracic surgery. However, because all patients received thoracic epidural analgesia, and because total epidural local anaesthetic consumption and rescue epidural boluses were not recorded, the independent analgesic contribution of pregabalin cannot be fully determined. Therefore, the observed advantages should be interpreted with appropriate caution. Future multicentre studies with larger sample sizes, longer follow-up for chronic post-thoracotomy pain, standardized assessment of epidural analgesic requirements, and evaluation of optimal dosing strategies are warranted to better define the long-term efficacy and safety of pregabalin within enhanced recovery and multimodal analgesia protocols for thoracic surgery.

## Ethics

**Ethics Committee Approval:** Ethical approval was obtained from the Institutional Ethics Committee of King George’s Medical University, U.P., (approval no: VI-PGTSC-IIA/P29, date: 27.10.2021).

**Informed Consent:** Written informed consent was obtained from all participants prior to enrolment.

## Figures and Tables

**Table 1. Baseline Demographic and Clinical Characteristics of Patients in Group I and Group II:** 

**Variables**		**Group I**	**Group II**
**Age**		37.72±13.92	38.49±15.69
**Gender**	**Female**	14 (35.9%)	18 (46.2%)
	**Male**	25 (64.1%)	21 (53.8%)
**ASA**	**Grade** **I**	24 (61.5%)	26 (66.7%)
	**Grade** **II**	15 (38.5%)	13 (33.3%)

ASA, American Society of Anesthesiologists

**Table 2. Comparison of Perioperative Hemodynamic Parameters Between Group I and Group II:** 

**Variables**		**Group I**	**Group II**	**t value**	***P* value**
**Heart** **rate** **(beats** **per** **minute)**	**Pre-op**	84.92±12.01	84.26±8.70	0.281	0.780
	**15** **min**	86.13±11.00	84.59±7.82	0.712	0.479
	**60** **min**	86.00±9.67	85.31±6.62	0.369	0.713
	**150** **min**	86.00±8.34	85.10±5.98	0.546	0.587
**Systolic** **BP** **(mmHg)**	**Pre-op**	121.82±8.77	123.03±8.56	-0.614	0.541
	**15** **min**	119.59±8.41	124.08±10.39	-2.096	0.039
	**60** **min**	121.77±7.47	120.59±8.17	0.665	0.508
	**150** **min**	120.33±7.88	122.38±7.90	-1.148	0.254
**Diastolic** **BP** **(mmHg)**	**Pre-op**	77.62±8.67	78.05±7.60	-0.236	0.814
	**15** **min**	77.15±7.89	77.92±7.02	-0.455	0.650
	**60** **min**	76.15±7.27	78.38±7.13	-1.368	0.175
	**150** **min**	76.15±6.30	77.87±6.49	-1.186	0.239

BP, blood pressure

**Table 3. Comparison of Postoperative Pain Intensity and Sleep Interference Between Group I and Group II:** 

**Variables**		**Group I**	**Group II**	**t value**	***P* value**
**Level** **of** **pain** **(numerical** **rating** **scale)**	**2** **h**	1.79±0.89	1.64±0.78	0.037	0.970
	**6** **h**	1.38±0.75	1.38±0.49	0.154	0.878
	**12** **h**	1.28±0.60	1.08±0.62	1.454	0.146
	**24** **h**	1.23±0.48	1.18±0.39	0.581	0.561
**Sleep** **interference** **rate** **score**	**SIR** **01**	12 (30.8%)	14 (35.9%)	0.241	0.886
	**SIR** **02**	18 (46.2%)	17 (43.6%)		
	**SIR** **03**	9 (23.1%)	8 (20.5%)		

**Table 4. Comparison of Rescue Analgesic Requirement and Opioid-sparing Effect (0-24 h):** 

**Variable**	**Group I (n = 39)**	**Group II (n = 39)**	**Test value**	***P* value**
**Patients** **requiring** **rescue** **analgesia,** **n** **(%)**	18 (46.2%)	27 (69.2%)	χ^2^=4.14	0.042
**Time** **to** **first** **rescue** **analgesia** **(hours)**	7.6±3.1	5.4±2.8	t=3.31	0.001
**Total** **opioid** **consumption** **in** **first** **24** **h** **(morphine** **equivalents,** **mg)**	6.2±3.0	8.6±3.4	t=-3.33	0.001
**Number** **of** **rescue** **doses** **in** **24** **h** **[median** **(IQR)]**	1 (0-2)	2 (1-3)	-	0.008

IQR, interquartile range

**Table 5. Postoperative Pain Intensity at Rest vs. Movement (NRS):** 

**Pain assessment**	**Time point**	**Group I (mean ± SD)**	**Group II (mean ± SD)**	**t value**	***P* value**
**Pain** **at** **rest**	2 h	1.79±0.89	1.80±0.78	0.06	0.95
	6 h	1.38±0.75	1.38±0.49	0.15	0.878
	12 h	1.28±0.60	1.08±0.62	1.45	0.146
	24 h	1.23±0.48	1.18±0.39	0.58	0.561
**Pain** **on** **deep** **breathing**	2 h	2.64±0.91	3.08±0.88	-2.17	0.033*
	6 h	2.18±0.84	2.62±0.79	-2.4	0.019*
	12 h	1.97±0.73	2.28±0.77	-1.82	0.073
	24 h	1.69±0.62	1.90±0.64	-1.48	0.143
**Pain** **on** **coughing**	2 h	3.10±0.96	3.74±0.93	-3	0.004*
	6 h	2.72±0.89	3.26±0.90	-2.64	0.010*
	12 h	2.33±0.78	2.71±0.82	-2.09	0.040*
	24 h	2.00±0.70	2.18±0.73	-1.12	0.265

test used- student's t test *: P value significant is less than 0.05

NRS, numerical rating scale; SD, standard deviation

**Table 6. Sedation and Neurocognitive Adverse Effects (0-24 h):** 

**Variable**	**Group I (n = 39)**	**Group II (n = 39)**	**Test value**	***P*** **value**
Sedation score (ramsay) at 2 h	2.38±0.54	2.10±0.45	t=2.50	0.015
Sedation score (ramsay) at 6 h	2.15±0.48	2.05±0.44	t=0.95	0.344
Excessive sedation requiring observation, n (%)	4 (10.3%)	1 (2.6%)	χ^2^=1.90	0.168
Dizziness/vertigo, n (%)	9 (23.1%)	3 (7.7%)	χ^2^=3.56	0.059
Somnolence, n (%)	7 (17.9%)	2 (5.1%)	χ^2^=3.11	0.078
Confusion/disorientation, n (%)	2 (5.1%)	0 (0%)	χ^2^=2.05	0.152
Visual disturbance, n (%)	1 (2.6%)	0 (0%)	χ^2^=1.01	0.315
